# Supplementary material for: Knowledge, attitude, and practice toward coronary heart disease secondary prevention among coronary heart disease patients in Shanghai, China
Source: PLoS One. 2025 Jan 22;20(1):e0316022. doi: 10.1371/journal.pone.0316022 (PMC11753668; doi:10.1371/journal.pone.0316022)
Supplement: S1 File — (DOCX) [file pone.0316022.s001.docx]

| Questionnaire ID： |
| --- |
| Dear Participant,  We are researchers from the Yangpu District Central Hospital in Shanghai and sincerely invite you to participate in our study. This research aims to understand the knowledge, attitudes, and practices (KAP) of patients with coronary heart disease (CHD) regarding CHD and its risk factors. The findings will provide a basis for developing scientific intervention strategies, which may help improve the health outcomes of others in the future. Participation in this study is entirely voluntary. The study has been reviewed and approved by the Ethics Committee. If you agree to participate, please refer to the following instructions.   1. Please complete the questionnaire. There are no right or wrong answers; simply respond based on your actual situation. If you encounter any questions during the process, feel free to reach out to us. Upon completion, kindly submit the questionnaire promptly. 2. This study involves a simple questionnaire survey and will not cause any harm to your physical or mental health. However, it may include some personal information, such as your gender and age. Please rest assured that your information will be kept strictly confidential and will not be disclosed. 3. As a participant, you are welcome to inquire about any information or progress related to this study. If you decide to withdraw from the study, please inform us, and your data will not be included in the research results.   Finally, we sincerely thank you for taking the time to support our scientific research!  □I acknowledge and consent to the use of the collected data for scientific research purposes.  Signature of informed consent：  Date of participation：Year___Month___Day |

| **Part 1: Basic Information** | | |
| --- | --- | --- |
| **Gender** | a. Male | b. Female |
| **Age** | | |
| **Residential area** | a. Rural  b. Urban  c. Suburban | |
| **Education** | a. Primary school or below b. Junior high school c. High school/vocational school d. Associate degree/bachelor's degree e. Master's degree or above | |
| **Job type** | a. Formal Employee/Occupation  b. Part-time c. Self-employed d. Unemployed e. Homemaker f. Student  h. Retired  g. Other | |
| **Monthly per capita income, RMB** | a.<2000  b.2000-5000  c.5000-10000  d.10000-20000  e.>20000 | |
| **Marital status** | a. Unmarried  b. Married  c. Divorced  d. Widowed | |
| **Smoking status** | a. Never smoker  b. Former smoker  c. Current smoker | |
| **Drinking status** | a. Never drinker  b. Former drinker  c. Current drinker | |
| **Comorbidities** | a. Hypertension  b. Diabetes  c. Thyroid disease d. Kidney disease e. Other | |
| **PCI times** |  | |

| **Part 2: Knowledge of Coronary Heart Disease and Its Risk Factors** | | | |  |
| --- | --- | --- | --- | --- |
| **Symptoms of coronary heart disease** |  |  |  | |
| **(1) Chest pain (angina)** | a. Familiar | b. Somewhat familiar | c. Unfamiliar | |
| **(2) Chest tightness** | a. Familiar | b. Somewhat familiar | c. Unfamiliar | |
| **(3) Shortness of breath** | a. Familiar | b. Somewhat familiar | c. Unfamiliar | |
| **(4) Pale skin, cold sweat** | a. Familiar | b. Somewhat familiar | c. Unfamiliar | |
| **(5) Dizziness** | a. Familiar | b. Somewhat familiar | c. Unfamiliar | |
| **(6) Palpitations** | a. Familiar | b. Somewhat familiar | c. Unfamiliar | |
| **(7) Confusion** | a. Familiar | b. Somewhat familiar | c. Unfamiliar | |
| **Risk Factors for Coronary Heart Disease** |  |  |  | |
| **(1) Smoking** | a. Familiar | b. Somewhat familiar | c. Unfamiliar | |
| **(2) Alcohol consumption** | a. Familiar | b. Somewhat familiar | c. Unfamiliar | |
| **(3) Diabetes** | a. Familiar | b. Somewhat familiar | c. Unfamiliar | |
| **(4) High level of low-density lipoprotein cholesterol** | a. Familiar | b. Somewhat familiar | c. Unfamiliar | |
| **(5) Poor control of hypertension (blood pressure >140/90 mmHg)** | a. Familiar | b. Somewhat familiar | c. Unfamiliar | |
| **(6) Obesity** | a. Familiar | b. Somewhat familiar | c. Unfamiliar | |
| **(7) Lack of physical activity** | a. Familiar | b. Somewhat familiar | c. Unfamiliar | |
| **(8) Family history of coronary heart disease** | a. Familiar | b. Somewhat familiar | c. Unfamiliar | |
| **(9) High-salt, high-sugar, high-fat diet** | a. Familiar | b. Somewhat familiar | c. Unfamiliar | |
| **(10) High psychological stress** | a. Familiar | b. Somewhat familiar | c. Unfamiliar | |

| **Part 3: Attitude towards Coronary Heart Disease Secondary Preventions** | | | | | |
| --- | --- | --- | --- | --- | --- |
| **Quitting smoking is crucial for controlling coronary heart disease.** | a. Strongly agree | b. Agree | c. Neutral | d. Disagree | e. Strongly disagree |
| **Quitting drinking is crucial for controlling coronary heart disease.** | a. Strongly agree | b. Agree | c. Neutral | d. Disagree | e. Strongly disagree |
| **Controlling blood glucose levels is crucial for controlling coronary heart disease.** | a. Strongly agree | b. Agree | c. Neutral | d. Disagree | e. Strongly disagree |
| **Controlling blood pressure is crucial for controlling coronary heart disease.** | a. Strongly agree | b. Agree | c. Neutral | d. Disagree | e. Strongly disagree |
| **Controlling blood lipid levels is crucial for controlling coronary heart disease.** | a. Strongly agree | b. Agree | c. Neutral | d. Disagree | e. Strongly disagree |
| **Controlling weight is crucial for controlling coronary heart disease.** | a. Strongly agree | b. Agree | c. Neutral | d. Disagree | e. Strongly disagree |
| **Maintaining a healthy diet is crucial for controlling coronary heart disease.** | a. Strongly agree | b. Agree | c. Neutral | d. Disagree | e. Strongly disagree |
| **Moderate exercise is crucial for controlling coronary heart disease.** | a. Strongly agree | b. Agree | c. Neutral | d. Disagree | e. Strongly disagree |
| **Maintaining a positive mentality is crucial for controlling coronary heart disease.** | a. Strongly agree | b. Agree | c. Neutral | d. Disagree | e. Strongly disagree |
| **Regular medication is crucial for controlling coronary heart disease.** | a. Strongly agree | b. Agree | c. Neutral | d. Disagree | e. Strongly disagree |
| **Regular follow-up visits are crucial for controlling coronary heart disease.** | a. Strongly agree | b. Agree | c. Neutral | d. Disagree | e. Strongly disagree |
| **Concerned that your coronary heart disease is worsening.** | a. Strongly agree | b. Agree | c. Neutral | d. Disagree | e. Strongly disagree |

| **Part 4: Practices Regarding Coronary Heart Disease and Its Risk Factors** | | | | | |
| --- | --- | --- | --- | --- | --- |
| **Take medication on time every day** | a. Always | b. Often | c. Sometimes | d. Rarely | e. Never |
| **Regularly go to the hospital for medical treatment** | a. Always | b. Often | c. Sometimes | d. Rarely | e. Never |
| **Seek medical treatment in a timely manner when experiencing discomfort such as chest pain or tightness** | a. Always | b. Often | c. Sometimes | d. Rarely | e. Never |
| **How frequently do you engage in the following behaviors?** |  |  |  |  |  |
| **(1) Smoking** | a. Every day | b. More than 3 times a week | c. 1-2 times a week | d. More than 2 times a month | e. Almost never |
| **(2) Drinking** | a. Every day | b. More than 3 times a week | c. 1-2 times a week | d. More than 2 times a month | e. Almost never |
| **(3) Measure blood glucose** | a. Every day | b. More than 3 times a week | c. 1-2 times a week | d. More than 2 times a month | e. Almost never |
| **(4) Measure blood pressure** | a. Every day | b. More than 3 times a week | c. 1-2 times a week | d. More than 2 times a month | e. Almost never |
| **(6) Exercise** | a. Every day | b. More than 3 times a week | c. 1-2 times a week | d. More than 2 times a month | e. Almost never |
| **(7) Low-salt, low-sugar, low-fat diet** | a. Every day | b. More than 3 times a week | c. 1-2 times a week | d. More than 2 times a month | e. Almost never |
| **(8) Maintain a good mentality** | a. Every day | b. More than 3 times a week | c. 1-2 times a week | d. More than 2 times a month | e. Almost never |
